# A concept elicitation study to understand the relationship between sleep and pain in rheumatoid arthritis and axial spondyloarthritis

**DOI:** 10.1007/s11136-023-03524-9

**Published:** 2023-10-27

**Authors:** Kimberly Raymond, Wen-Hung Chen, Marguerite Bracher, April Foster, Andrew Lovley, Cory Saucier, Kristi Jackson, Eleanor J. McDermott

**Affiliations:** 1 QualityMetric Incorporated, Johnston, RI USA; 2 GSK, Global Value Evidence and Outcomes, 1250 South Collegeville Road, Building 4, 4th floor, Collegeville, PA 19426 USA; 3 GSK, Global Value Evidence and Outcomes, Stevenage, UK; 4 GSK, Real-World Study Delivery, Stevenage, UK

**Keywords:** Rheumatoid arthritis, Axial spondyloarthritis, Sleep disturbance, Pain, Qualitative study, Patient perspective

## Abstract

**Purpose:**

This qualitative study (GSK study: 213635) was designed to better understand sleep disturbance as experienced by individuals with rheumatoid arthritis (RA) or axial spondyloarthritis (axSpA), and the relationship between sleep disturbance and pain and other aspects of the disease and disease activity.

**Methods:**

Sixty-minute, one-on-one, concept elicitation interviews were conducted with 30 participants (15 with RA and 15 with axSpA) from the US. Interviews were audio-recorded and transcribed verbatim. Interview transcripts were coded and analyzed to explore themes related to pain and sleep disturbance, and relationships among those themes.

**Results:**

Pain was a prominent driver of sleep disturbance; 12 participants with RA (80%) and 14 with axSpA (93%) reported that pain impacted their ability to fall asleep, while all 15 with RA (100%) and 14 with axSpA (93%) reported that pain impacted their ability to stay asleep. Two-thirds of participants with RA (67%) or axSpA (60%) described a bi-directional relationship, whereby pain worsened sleep disturbance and sleep disturbance further aggravated pain. Factors other than pain, such as fatigue and emotional health, were also reported as important contributors to sleep disturbance (RA: *n* = 12/15, 80%; axSpA: *n* = 14/15, 93%). Participants with RA or axSpA described complex interconnections between fatigue, emotional health, pain, and sleep, often labeling these relationships as “*vicious cycles*”. Notably, half of all participants reported sleep disturbance occurring without pain or other understood causes.

**Conclusion:**

These perspectives collected from people with RA or axSpA suggest that reducing sleep disruption directly may offer clinically relevant benefits.

**Supplementary Information:**

The online version contains supplementary material available at 10.1007/s11136-023-03524-9.

## Plain English summary

Disturbed sleep is a major concern for people with rheumatoid arthritis (RA) or axial spondyloarthritis (axSpA) and can have a major effect on their health-related quality of life. This study aimed to better understand the interaction between sleep and pain in these conditions, as well as any other factors that may impact sleep. Thirty people with RA or axSpA were asked directly to describe their own experience with sleep disturbance, pain, fatigue, and emotional health. Most people considered pain to be a significant cause of disturbed sleep, although the relationship between pain and sleep often varied; sometimes disturbed sleep could make disease-related pain worse, and sleep disruption could occur regardless of pain or other understood causes. In addition, many of the people interviewed described disease-related factors other than pain that they felt were responsible for disrupting sleep, such as fatigue and anxiety. These perspectives from people living with RA or axSpA suggest that healthcare providers should consider and potentially manage all aspects of disturbed sleep during appointments with their patients, and not just their patients’ level of pain.

## Introduction

Rheumatoid arthritis (RA) and axial spondyloarthritis (axSpA) are common autoimmune rheumatic diseases [1, 2]. RA is characterized by pain, swelling, and stiffness in multiple joints [2] while axSpA is predominantly characterized by inflammation of the sacroiliac joints and spine, often causing back pain [2, 3]. Patients with axSpA are classified as having either radiographic (r-axSpA, or ankylosing spondylitis [AS]) or non-radiographic (nr-axSpA) disease depending on disease features [4].

The disease burden for patients with RA or axSpA is substantial, leading to impaired health-related quality of life (HRQoL) in terms of physical, emotional, and social well-being compared with the general population [2, 5–12]. RA can lead to painful joint degeneration, functional impairment, and morbidity, and disability is common and substantial [2]; in a large US study, 35% of participants with RA had employment-related disability after 10 years [6]. Similarly, patients with axSpA often experience impairment or loss of physical function [10]. Across both conditions, pain is often highlighted as one of the most unpleasant symptoms and persists even among those with well-controlled disease [12–17]. Known risk factors for worse pain include poor mental health, poor physical function, and presence of comorbidities [13, 17–19]. The common goals of disease management are to reduce and/or control inflammation and pain, reduce functional limitations, maintain social relationships and work ability, and decrease complications [7, 10].

Sleep disturbance is common, and a major concern of patients with RA or axSpA [20–22]. Poor sleep has been widely associated with impairing physical activity and other daily functions [1, 8, 21–26]. It also exacerbates systemic inflammation (including increased circulating concentrations of tumor necrosis factor and interleukin-6) and pain sensitivity, mental and physical fatigue, and mood disorders such as depression, further deteriorating the HRQoL of people with rheumatoid conditions [27–31].

Several potential confounding factors exist for sleep disturbance in patients with rheumatic conditions, including pain [27, 28]. Previous research suggests that a complex relationship exists between sleep and pain in patients with RA or axSpA [28, 30]. However, despite evidence regarding the presence of this relationship, the direction of causality and mechanisms involved are not clear [32]; while joint (RA) and back (axSpA) pain can disrupt sleep, studies in both conditions have demonstrated a reverse relationship, where disturbances in sleep can also aggravate pain [23, 31, 33]. In addition, other disease-related factors of RA and axSpA, independent of pain (such as fatigue, depression, anxiety, stress, and inflammatory markers), have also been associated with sleep disturbance [24, 31, 34–37].

This study aimed to better understand sleep disturbance as experienced directly by individuals with RA or axSpA, as it relates to pain and other aspects of the disease and disease activity, through descriptive evidence gathered from qualitative concept elicitation (CE) interviews. Aspects of sleep disturbance that occur independently of pain from a patient perspective, and which could be potential therapeutic targets benefiting patients’ well-being, were also explored.

## Methods

### Study design

This was a qualitative, non-interventional, cross-sectional study (GSK study: 213635). Sixty-minute CE interviews were conducted with 30 participants (15 with RA and 15 with axSpA) from the US.

### Participants

Participants were recruited and screened in collaboration with a third-party recruitment specialist using pre-existing patient panels, recruiter databases, physician referrals, and a patient organization (Spondylitis Association of America).

Eligible participants were required to have moderate or severe RA (diagnosis received over the age of 18 years and at least 2 years prior to screening) or axSpA (either radiographic or non-radiographic), be aged ≥ 18 years old, be fluent in English, and provide a physician note prior to interview confirming their diagnosis, disease severity, and current prescribed medication. Those with RA must have scored ≥ 4 on a pain numeric rating scale (NRS) in the past 7 days as assessed during screening, while those with axSpA must have scored ≥ 4 on the Bath Ankylosing Spondylitis Disease Activity Index and rated their back pain ≥ 4 on a 0–10 NRS in the past 7 days. All participants reported sleep problems in the past 14 days during screening (e.g., woke up in the middle of the night and could not fall back to sleep; woke up early in the morning; had trouble initiating and maintaining sleep). These sleep problems were not required to be specific to their condition.

Participants were originally excluded if they had a history of other inflammatory rheumatologic or systemic autoimmune disorder, other than Sjögren’s syndrome secondary to RA or axSpA. This was subsequently adjusted for those with axSpA due to difficulties in recruiting individuals who did not have a comorbid inflammatory condition. Therefore, axSpA participants were eligible if they had a history of mixed connective tissue disease, systemic lupus erythematosus, scleroderma, Crohn’s disease, ulcerative colitis, or RA. For those with axSpA and comorbid RA, restrictions were set to include only those who had not experienced RA-related pain in the past 7 days.

Given the overlap in the experiences of RA and axSpA [38], a total of 30 participants (15 with each condition) was determined by saturation analysis (see “Data analysis” section) to be sufficient to capture all relevant information related to sleep and pain in the two conditions. While no specific participant quotas other than a diagnosis of RA or axSpA were set, purposive sampling [39–41] was used to recruit a diverse participant group in terms of geographic region, sex, and race/ethnicity. All participants provided written, informed consent prior to their scheduled interview.

### Qualitative CE interviews

One-on-one CE interviews were conducted (via telephone or video call) by trained qualitative researchers using a semi-structured interview guide [42]. The guide was developed using findings from a review of published literature related to sleep disturbance in RA and axSpA and subsequently finalized following input from a panel of five people with RA from the US.

Interviews began with a brief overview of the study, and the participant provided consent to audio-record the interview. This was followed by introductory questions intended to provide background information and establish rapport with the participant. Next, the interviewer asked a series of questions designed to elicit descriptions of participants’ experience with sleep disturbances associated with their RA or axSpA. Specifically, questions explored potential causes of sleep disturbances at times when participants did and did not experience pain.

Although conversations were guided by the semi-structured interview guide, interviewers also used their discretion to further probe different aspects of participants’ responses (for both elaboration and clarification). Combining the semi-structured interview guide and ad hoc probing allowed the study team to gather detailed descriptions of participants’ experience with sleep disturbance related to their condition. Interview recordings were subsequently transcribed verbatim for concept coding and analysis.

### Data analysis

Interview transcripts were coded using NVivo software (v.12; QSR International Pty Ltd., 2018) and analyzed using a combination of thematic and content analysis to explore themes related to pain and sleep disturbance, and to analyze relationships among those themes [43, 44]. Transcripts were first reviewed in full for familiarity; they were then read again and considered line by line. During this process, relevant text was assigned to a code that described the content being coded.

Content was analyzed and (as appropriate) text assigned to a set of a priori codes (i.e., codes developed in advance and linked to the questions in the interview guide). These codes were based on concepts of interest to the research question (e.g., sleep, pain, fatigue). Additional themes of interest (not pre-determined) were also identified in the text using thematic analysis and assigned to new codes [45]. All codes were iteratively refined and grouped, as appropriate, into larger themes representing similar ideas.

Relationship codes were also developed and used to capture links between concepts. For example, if participants noted that pain influenced their sleep, content would be coded to “pain”, and “influence on sleep”. Once all data were coded, themes were analyzed and matrices were run to identify and examine relationships among concepts, including directional relationships.

Saturation analyses [46] were conducted to confirm the size of the participant groups was adequate to fully explore concepts of interest, and that additional interviews would not have resulted in the identification of new concepts or relationships for either the RA or axSpA participants.

Several strategies were employed to pursue the trustworthiness of our analyses. The members of the research team are trained extensively in qualitative research, and were fully briefed on the research questions and goals of the current study prior to commencing data collection. The varied experiences and professional backgrounds of the study team were considered during training to promote data collection and interpretation that avoided bias from prior assumptions. Researcher triangulation and constant comparison was used to review interviewer field notes, coding, and analyses, to promote fidelity of data collection and integrity, and to reduce the possibility that any single researcher’s viewpoint was privileged over that of others. Credibility was bolstered through interview debriefing meetings to assess and promote congruence across interviewers, and to promote collaboration of perspective. Coders also met regularly to discuss interpretations and alternative explanations before determining the findings. Dependability was partly pursued with coder reliability, as two researchers independently coded the first three transcripts and then met with the principal investigator to discuss any discrepancies and reach consensus, before independently coding the remaining transcripts. Discrepancies and decisions taken were documented, including adjustments to the code book, and ad hoc meetings were held throughout the coding process to discuss concerns. Regarding confirmability, the research team generated a detailed audit trial of methodological decisions, coding guidelines, and query output to allow for replicability. The use of purposive sampling and the inclusion of a diverse sample increased the likelihood that findings could be applicable and transferable to the larger population of individuals with these conditions.

## Results

### Participant characteristics

A total of 30 participants (RA = 15; axSpA = 15) were recruited for CE interviews (Table 1). The mean age was 48.4 years (RA, 55.3 years; axSpA, 41.6 years). Most were female (*n* = 24/30, 80%) and self-identified as White (*n* = 21/30, 70%). The majority of those with axSpA reported that their disease was confirmed radiographically (*n* = 13/15, 87%).

**Table 1:** Participant characteristics at screening

Characteristic	RA (*n* = 15)	axSpA (*n* = 15)
Age range (years), *n* (%)		
18–29	0 (0)	3 (20)
30–49	3 (20)	8 (53)
50–69	12 (80)	3 (20)
70 +	0 (0)	1 (7)
Sex, *n* (%)		
Male	3 (20)	3 (20)
Female	12 (80)	12 (80)
Race, *n* (%)		
White	10 (67)	11 (73)
Black/African American	4 (27)	2 (13)
Asian	0 (0)	2 (13)
Mixed race	1 (7)	0 (0)
Physician-reported disease severity^a^, *n* (%)		
Moderate	9 (60)	10 (67)
Moderate–severe	2 (13)	0 (0)
Severe	4 (27)	5 (33)
Level of pain due to RA/axSpA in past 7 days (NRS 1–10^b^), *n* (%)		
4	3 (20)	0 (0)
5	1 (7)	6 (40)
6	4 (27)	0 (0)
7	1 (7)	2 (13)
8	2 (13)	5 (33)
9	2 (13)	2 (13)
10	2 (13)	0 (0)
Experience of pain due to RA/axSpA daily, *n* (%)		
Yes	7 (47)	12 (80)
Experienced sleep problems in past 14 days, *n* (%)		
A little	6 (40)	1 (7)
Somewhat	4 (27)	5 (33)
Quite a bit	4 (27)	4 (27)
Very much	1 (7)	5 (33)
Experience of fatigue daily, *n* (%)		
Yes	8 (53)	8 (53)

^a^All participants provided written confirmation from a physician of their positive diagnosis, severity level, and prescribed medications

^b^1–10 NRS with a higher value indicating worse pain

*axSpA* axial spondyloarthritis, *NRS* numeric rating scale, *RA* rheumatoid arthritis

When asked to rate their level of pain due to RA/axSpA in the past 7 days (using a 1–10 NRS with a higher value indicating worse pain), over half of participants (*n* = 16/30, 53%) reported experiencing a pain level of ≥ 7 (7/15 [47%] those with RA and 9/15 [60%] with axSpA). Most participants with RA/axSpA experienced sleep problems “somewhat” or “quite a bit” in the past 14 days, and over half (RA, *n* = 8/15 [53%]; axSpA, *n* = 8/15 [53%]) reported daily fatigue.

### Descriptive experience of sleep disturbance and related concepts

Participants provided descriptive information throughout the CE interviews on their experience of sleep, as well as three individual concepts known to be related to sleep disturbance (pain, fatigue, and emotional health [e.g., stress, anxiety, and depression). The subsequent section of the results describes the way participants discussed these concepts as they relate to their experience of sleep. Examples of relevant patient quotes are included in Table 2 and Supplementary Table 1.

**Table 2:** Example quotes provided by participants in describing their experience of sleep and sleep quality, pain, fatigue, and emotional health

RA	axSpA
Sleep	
“I’m in the bed for at least eight or nine hours, but I probably only sleep five or six hours, because the rest of the time I’m turning—I’m uh, twisting and turning, and something feels uncomfortable, or I don’t know. I just—I don’t sleep very well.” (Pt 04, RA)	“So, um, I am, I’m in the bed for eight hours. Um, but as far as like sleep where I, I know that I’m asleep, it’s maybe five of those, I would guess, um, because I wake often, um, yeah. I mean, where I’m aware of it, and then I fall back asleep.” (Pt 08, axSpA)
Pain	
“It’s in different locations. You can feel it over your entire body, but usually it’s symmetrical. So, whatever side—if your elbow is hurting on the right side, then your knee is going to be hurting—you know, it’s like that. It doesn’t—it’s not like one thing on this side and one thing on that side. It’s—it’s all the way down.” (Pt 04, RA)	“In the back, it feels like an ache as well. It’s not—I never experienced super sharp pain unless it’s like going—it’s starting to travel to other areas. I can like vividly feel it. Uhm, it feels dull in my back as well. A dull ache, but, uhm, more like I have to like crack my back or like push in certain spots.” (Pt 07, axSpA)
Fatigue	
“I experience it [fatigue] every single day, but some days it might be like, down on a level three; other days it could be up to eight or nine.” (Pt 14, RA)	“I’ve known tiredness, it seems like tiredness is not the same as fatigue. Fatigue is more when your whole body is, is kind of shutting down. And tiredness is when you need rest and sleep, and it’s a different thing than fatigue.” (Pt 03, axSpA)
Emotional health	
“Like the anxiety part of it, it’s a physiological thing that I learned about my body that when I’m experiencing like, it’s not like I’m anxious, like oh my god, I’m going to die. It literally is a physiological reaction to my body being like either severe pain or sick. It just happens.” (Pt 01, RA)	“Yeah, because it’s a stress. I’m already stressed. Stress probably causes inflammation, I don’t know but yeah and none—none of this crap was really going on when it all first began, at least—at least not to this level, let’s put it that way but it is now.” (Pt 05, axSpA)

*axSpA* axial spondyloarthritis, *Pt* patient, *RA* rheumatoid arthritis

#### Sleep

Many participants with RA (*n* = 11/15, 73%) reported not feeling well rested on most days. The majority (*n* = 9/15, 60%) reported regularly tossing and turning, and that the number of hours of sleep per night varied (*n* = 10/15, 67%), with total hours ranging from 2.5 to 10 h per night. Consistent difficulty falling asleep was described by three participants (20%).

Among those with axSpA, most (*n* = 14/15, 93%) reported not getting enough sleep and feeling exhausted throughout the day. Eleven participants (73%) reported difficulties falling asleep. Sleep ranged from 3.5 to 10 h per night.

#### Pain

Pain was reported by participants with RA as the most bothersome symptom (*n* = 9/15, 60%), having the most impact on their life (*n* = 8/15, 53%). Many noted that their pain varied in intensity (*n* = 11/15, 73%), frequency (*n* = 8/15, 53%), and by bodily location, including upper extremity (e.g., hands/fingers, wrists, elbows, shoulders, and neck) and/or lower extremity (e.g., hips, knees, ankles, and feet). Participants with RA also said that pain was triggered/worsened by factors such as climate, diet, and physical exercise, and was worse and less manageable during a flare-up.

Pain was reported by 40% of those with axSpA (*n* = 6/15) as the most bothersome symptom associated with their condition, and by 60% (*n* = 9/15) as having the most impact on their life. Most reported pain as varying in intensity (*n* = 10/15, 67%), and all 15 (100%) described having good and bad days when pain was more or less tolerable. Most participants with axSpA (*n* = 12/15, 80%) noted that they experienced pain every day. In terms of bodily location, those with axSpA described pain mostly in their lower back and spine, although pain could radiate to the neck, shoulders, arms, legs, hands, and feet. Similar to the experience of those with RA, pain was worse and less manageable during a flare-up.

#### Fatigue

Participants with RA described their fatigue as exhaustion, draining, and feeling tiredness but not necessarily sleepiness. Fatigue often fluctuated (*n* = 6/15, 40%), with over half (*n* = 8/15, 53%) experiencing fatigue every day. They also described that fatigue was worse during a flare-up. The climate, physical activity level, and stress regarding work or daily activities and the coronavirus disease 2019 (COVID-19) pandemic were described as triggers for their fatigue (*n* = 6/15, 40%).

Those with axSpA described their fatigue as a feeling of exhaustion, a lack of/low energy, and tiredness. Fatigue levels were reported as fluctuating or varying (*n* = 10/15, 67%), and as worse and less manageable during a flare-up. Participants with axSpA said physical activity drained their energy and worsened their fatigue, as did changing weather and stress from the COVID-19 pandemic (*n* = 9/15, 60%).

#### Emotional health

Most participants with RA (*n* = 14/15, 93%) described emotional health impacts in general, while a third (*n* = 5/15, 33%) reported emotional impacts specifically related to their RA, including feelings of stress or anxiety due to symptoms. Several (*n* = 6/15, 40%) emphasized their holistic approach to managing the disease; this included two participants who reported that by reducing stress (related or unrelated to RA) they could better manage RA symptoms such as pain and sleeplessness.

Similar to RA, nearly all (*n* = 14/15, 93%) participants with axSpA described emotional health impacts in general, while about half (*n* = 7/15, 47%) reported emotional impacts specifically related to their axSpA. Often, they described experiencing stress, anxiety, or feeling depressed due to their axSpA. Like participants with RA, those with axSpA emphasized managing stress to better manage the physical symptoms of the disease.

### Linear and bi-directional relationships between concepts

**Fig. 1:**
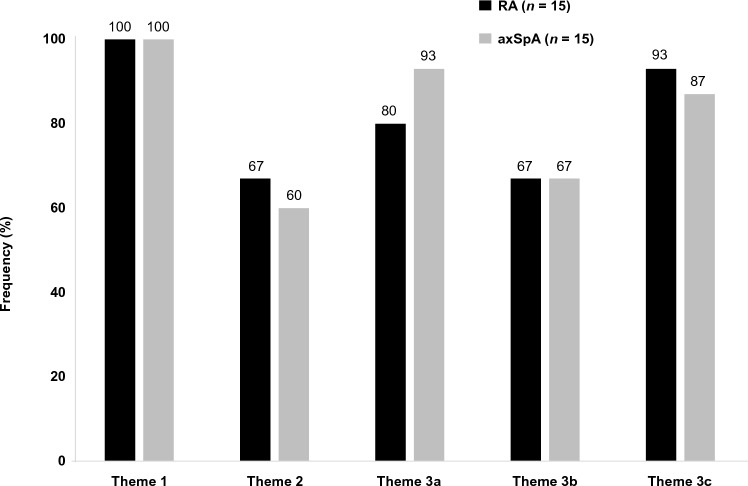
Identification of themes across participant interviews. Three themes were identified across the participant interviews: theme 1—pain as a primary driver of sleep disturbance, affecting both falling asleep and staying asleep; theme 2—a bi-directional relationship whereby pain worsens sleep disturbance and sleep disturbance further aggravates pain; theme 3—other factors influencing sleep disturbance, including sleep disturbance due to disease-related causes other than pain (e.g., anxiety, fatigue, stiff, locking joints) or other known aspects related to disease management (e.g., medication; theme 3a), sleep disturbance due to causes unrelated to disease (e.g., needing to use the restroom; theme 3b), and sleep disturbance due to unknown causes (theme 3c). *axSpA* axial spondyloarthritis, *RA* rheumatoid arthritis

Three themes were identified across the CE interviews (Fig. 1). Pain was a primary driver of sleep disturbance, affecting both falling asleep and staying asleep (theme 1). In addition, a bi-directional relationship was identified whereby pain worsened sleep disturbance and sleep disturbance further aggravated pain (theme 2). For theme 3, other factors influenced sleep disturbance, including sleep disturbance due to disease-related factors other than pain (e.g., anxiety, fatigue, stiff, locking joints) or other known aspects related to disease management such as medication (theme 3a); sleep disturbance due to causes unrelated to disease, such as needing to use the restroom (theme 3b); and sleep disturbance due to unknown causes (theme 3c). Examples of quotes relevant to each theme are shown for participants with RA and axSpA in Table 3 and Supplementary Tables 2 and 3.

**Table 3:** Example quotes provided by participants with RA and axSpA in relation to identified themes

Theme	Example quotes
Theme 1: Pain as a primary driver of sleep disturbance, affecting both falling asleep and night-time waking	“If I hurt I—I—I have a hard time going to sleep because of the ache and the hurt and it—I just can’t go to sleep.” (Pt 08, RA)
	“I mean, if the pain is worse, I’ll sleep less, and my fatigue is worse the next day. It’s as simple as that, for me” (Pt 01, axSpA)
Theme 2: A bi-directional relationship whereby pain worsens sleep disturbance, and sleep disturbance further aggravates pain	“I think they’re all related. I think that each one of them plays a role and if for one, I’m not sleeping well then I’d say it’s going to impact my emotions and probably my pain for the day the next day or even if the pain is impacting my life it’s going to affect my emotions and my sleep because I’m not going to be able to sleep and then all I’m trying to do is not hurt, so that’s going to affect my emotions, too. But I think they all intermix.” (Pt 08, RA)
	“Well, I think with that one is my sleep is worse when my pain is worse, and if my pain is bad, it can trigger a depressive, an episode of depression. Um, so the next day is just going to be more of the same, more pain, more fatigue.” (Pt 08, axSpA)
Theme 3: Other factors influencing sleep disturbance	“Then, the other, is just kind of the mental drag of having RA has on you too, Um, I think it’s those things that probably impact my sleep, um, when it’s not pain more than anything.” (Pt 04 RA)
	“If I’m, you know, dealing with depression or something like that, then definitely it’s hard to go to sleep because, you know, my mind is racing, I’m having a lot of thoughts. Um, and then when I wake up, instead of, uh, falling right back asleep when I wake up immediately, you know, my mind starts going. And it could be, um, frustration that kicks in, and then, you know, my mind goes to this different place, um, about, you know, either the pain or whatever it is, um, which makes it a little bit harder to fall back asleep.” (Pt 08, axSpA)

*axSpA* axial spondyloarthritis, *Pt* patient, *RA* rheumatoid arthritis

#### Theme 1: pain as a primary driver of sleep disturbance

**Fig. 2:**
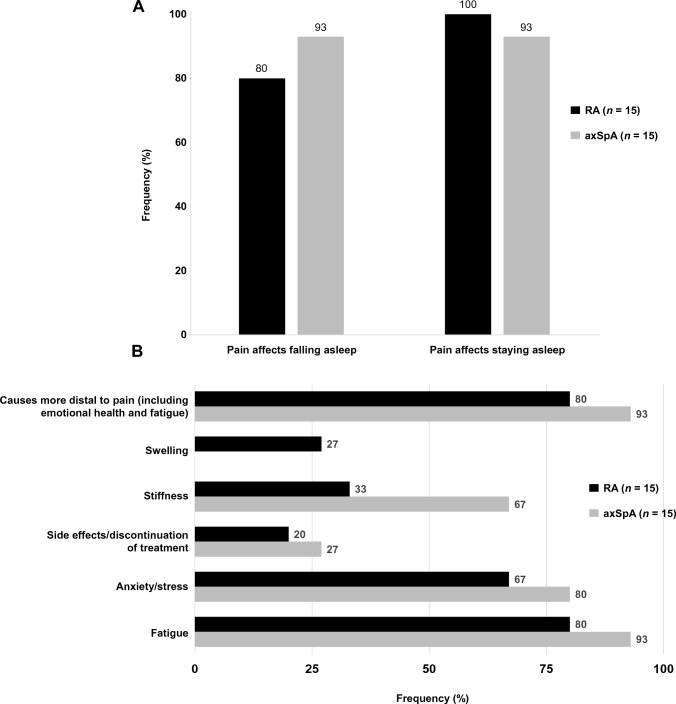
Impact of pain **A** and other factors **B** on sleep across participant interviews. *axSpA* axial spondyloarthritis, *RA* rheumatoid arthritis

Pain was most frequently implicated as the reason for sleep disturbance and was experienced by 100% (*n* = 30/30) of participants with RA or axSpA at some time. A substantial number reported difficulties with both falling and staying asleep due to pain (Fig. 2A); 12 with RA (80%) and 14 with axSpA (93%) reported that pain impacted their ability to fall asleep, and all 15 with RA (100%) and 14 with axSpA (93%) reported that pain impacted their ability to stay asleep.

#### Theme 2: bi-directional relationship between pain and sleep

Two-thirds of those with RA (*n* = 10/15, 67%) or axSpA (*n* = 9/15, 60%) described a bi-directional relationship, whereby pain worsened sleep disturbance and sleep disturbance further aggravated pain. Pain was often more manageable after sleeping well.

#### Theme 3a: sleep disturbance due to disease-related causes other than pain

Disease-related factors other than pain, such as fatigue and emotional health, were also important contributors to sleep disturbance (RA: *n* = 12/15, 80%; axSpA: *n* = 14/15, 93%; Fig. 2B).

Most participants with RA (*n* = 12/15, 80%) described a relationship between fatigue and sleep (Fig. 2B), in that the level of fatigue they experience on a given day impacted their ability to sleep on subsequent evenings. Some described a cycle of fatigue disrupting sleep and resulting in more fatigue, and conveyed frustration from not being able to sleep well at night even while experiencing fatigue or extreme exhaustion. Others described a different scenario in which poor sleep led to extreme fatigue and eventually facilitated their ability to sleep. Similarly, most participants with axSpA (*n* = 14/15, 93%) reported that fatigue impacted sleep in some way, negatively and/or positively. Some explained that when fatigue led to exhaustion, they often experienced better sleep, an easier time falling asleep, and slept longer than usual. In contrast, others with axSpA reported that fatigue could often make it more difficult to fall or stay asleep.

Emotional health, such as anxiety, influenced the ability to fall asleep for many participants with RA or axSpA, and in turn, left them more irritable, frustrated, angry, or depressed. Most RA participants (*n* = 14/15, 93%) and all with axSpA reported that when their sleep was poor, their emotional health was negatively impacted. Many participants with RA (*n* = 11/15, 73%) and axSpA (*n* = 8/15, 53%) described feeling more irritated, frustrated, sad, moody, less motivated, or anxious after not sleeping well. Six participants with RA (*n* = 6/15, 40%) reported feeling anxious or nervous about their inability to fall asleep; the hours spent thinking about falling asleep and then not being able to fall asleep further intensified problems with emotional health. Two-thirds of those with RA (*n* = 10/15, 67%) specifically reported that anxiety and stress impacted their ability to fall asleep (Fig. 2B). Similarly, those with axSpA (*n* = 12/15, 80%) reported that negative emotions impacted their sleep; they described that on days when they were feeling stressed (disease-related or not) their ability to fall asleep (*n* = 10/15, 67%) or stay asleep (*n* = 4/15, 27%) was affected.

Other disease aspects, such as swelling and stiffness, or medication, also affected sleep (Fig. 2B). Four participants with RA (27%) reported swelling interfering with their ability to fall or stay asleep, and five (33%) with RA and 10 (67%) with axSpA reported that stiffness could interfere with their ability to both fall and stay sleep. Three participants with RA (20%) and four with axSpA (27%) reported side effects of medication or discontinuing treatment for their RA/axSpA symptoms because of side effects impacting sleep.

#### Theme 3b: sleep disturbance due to causes unrelated to disease

Fourteen participants with RA (93%) described other factors that impacted their sleep that were unrelated to their condition, such as needing to use the restroom, age, menopause, climate, diet, caffeine intake, noise, symptoms related to comorbidities, and role (taking care of children or dogs). Similarly, nine participants with axSpA (60%) specifically reported other factors related to difficulty staying asleep, such as needing to use the restroom at night, noise, caffeine intake, alcohol intake, diet, climate, role (parent of young children), having a bad dream/nightmare, and muscle spasms. In addition, eight participants with axSpA (53%) reported that work or reading before bed, and comorbidity-related symptoms, led to difficulty falling asleep.

#### Theme 3c: sleep disturbance due to unknown causes

Half of all participants with RA/axSpA reported that there were times they had trouble sleeping (both falling asleep and waking), although they were unable to attribute the sleep disruption to anything specific (RA: *n* = 8/15, 53%; axSpA: *n* = 7/15, 47%). Most assumed or spoke of these occurrences as though they were related to some aspect of their disease, or a habit formed because of their disease.

## Discussion

This qualitative CE interview study found that pain was a prominent driver of sleep disturbance in participants with RA or axSpA, resulting in trouble both falling asleep and staying asleep. A bi-directional relationship, whereby pain worsened sleep disturbance and sleep disturbance further aggravated pain, was described by approximately two-thirds of all participants with RA or axSpA. In addition, other factors were identified as influencing sleep disturbance, including disease-related causes other than pain (e.g., anxiety, fatigue, stiff, locking joints) or other known aspects related to disease management (e.g., medication), causes unrelated to disease (e.g., needing to use the restroom), and unknown causes.

Sleep disturbance is common in patients with rheumatic conditions [27, 30]. It has been reported that over half (54.8%) of patients with AS experience sleep disturbance [20, 21], while the prevalence of sleep disturbance in patients with RA has been estimated at 60–80% compared with 10–30% in the general population [22]. While management of the underlying disease, specifically pain, has the potential to improve sleep outcomes for some patients, pain may not always be considered or well-treated in RA and axSpA for a variety of reasons, including misdiagnosis and delayed diagnosis, and therapy-related, physician-related, and patient-related factors [2, 47, 48]. Furthermore, several studies have shown that pain persists in many people with RA, despite commencing new treatments for their disease including tumor necrosis factor-α inhibitors [13].

In the current study, half of all participants with RA or axSpA reported sleep disturbance occurred without pain or other understood causes. This suggests that sleep disturbance may be a separate symptom of the disease, and/or prompted by other disease-related factors. In addition, many participants with RA or axSpA described factors other than pain that were responsible for disrupting sleep, such as fatigue and emotional health. Participants with RA or axSpA recognized and described similar complex interconnections between fatigue, emotional health, pain, and sleep. Although they often reported pain as the driver of the relationship between sleep, fatigue, and emotional health, they sometimes acknowledged uncertainty regarding which was the precipitating factor.

Efforts were taken to employ strategies to mitigate impacts of study limitations, although these limitations should be acknowledged. While an attempt was made to recruit a mix of participants with nr-axSpA and AS, specific quotas were not established and so most of those recruited had AS (87%). While no clear differences were noted in the responses of these two types of participants, it is possible that the sample with axSpA included in this study may have more advanced or severe disease and symptoms, including pain. Further studies that explore the relationship between pain and sleep in those with earlier disease (i.e., nr-axSpA) would be of interest.

Attempts were also made to recruit a demographically balanced study population, although participants included in this study were predominantly female. This reflects the higher prevalence of females diagnosed with rheumatic conditions in the broader population, with females three times more likely than males to be diagnosed with an autoimmune rheumatic condition [49]. There were no prominent differences noted in the experience of sleep and pain between males and females in this study.

Participants were eligible for this study if they experienced pain due to their condition in the past 7 days. Future studies may benefit from exploring sleep disturbance in those who do not experience pain as a point of comparison, and to better investigate sleep difficulty outside of pain.

Finally, interviews were conducted during the early stages of the COVID-19 pandemic which may have contributed to fatigue, emotional health, and sleep loss in study participants, in common with the general population [50–54].

The study also has some noteworthy strengths, including the use of a semi-structured interview consisting of open-ended questions in combination with ad hoc probing which was successful in capturing patients’ own perspectives of the relationship between sleep and pain and other factors. Participants with RA and axSpA recognized and described similar complex interconnections between sleep disturbance, pain, fatigue, and emotional health. In addition, they were able to identify non-disease-related factors that interrupted their sleep, confirming their ability to thoughtfully discern different reasons for their sleep disturbance. Overall, participants confirmed findings from existing literature by describing factors other than pain (e.g., fatigue and emotional health) that were sometimes responsible for their disrupted sleep.

## Conclusions

Pain was a significant driver of sleep disturbance, although interactions between pain and sleep among patients with RA and axSpA vary. Sleep disturbance could make disease-related pain worse, and disease‐related sleep disturbances could occur regardless of pain. These findings indicate that understanding sleep outcomes, as well as pain and other aspects of disease activity, is important for optimal management of RA and axSpA. While therapeutic efforts to reduce pain may have the most impact on improving sleep in some patients with RA and axSpA, reducing sleep disruption directly may also offer clinically relevant benefits independent of, and in addition to, improvements in pain. The future inclusion of a sleep disturbance measure in clinical trials may be beneficial to understand additional possible benefits of potential treatments for RA and axSpA.

### Supplementary Information

Below is the link to the electronic supplementary material.

Supplementary file1 (DOCX 35 KB)

## Data Availability

The datasets generated and/or analyzed during the current study are not publicly available but are available from the corresponding author on reasonable request.

## References

[1] Zhang L, Shen B, Liu S (2021). Rheumatoid arthritis is associated with negatively variable impacts on domains of sleep disturbances: Evidence from a systematic review and meta-analysis. Psychology, Health & Medicine.

[2] Mease PJ, Bhutani MK, Hass S, Yi E, Hur P, Kim N (2022). Comparison of clinical manifestations in rheumatoid arthritis vs. spondyloarthritis: A systematic literature review. Rheumatology and Therapy.

[3] Walsh JA, Magrey M (2021). Clinical manifestations and diagnosis of axial spondyloarthritis. Journal of Clinical Rheumatology.

[4] Rudwaleit M, van der Heijde D, Landewé R, Listing J, Akkoc N, Brandt J, Braun J, Chou CT, Collantes-Estevez E, Dougados M, Huang F, Gu J, Khan MA, Kirazli Y, Maksymowych WP, Mielants H, Sørensen IJ, Ozgocmen S, Roussou E, Valle-Oñate R, Weber U, Wei J, Sieper J (2009). The development of Assessment of SpondyloArthritis international Society classification criteria for axial spondyloarthritis (part II): Validation and final selection. Annals of the Rheumatic Diseases.

[5] Safiri S, Kolahi AA, Hoy D, Smith E, Bettampadi D, Mansournia MA, Almasi-Hashiani A, Ashrafi-Asgarabad A, Moradi-Lakeh M, Qorbani M, Collins G, Woolf AD, March L, Cross M (2019). Global, regional and national burden of rheumatoid arthritis 1990–2017: A systematic analysis of the Global Burden of Disease study 2017. Annals of the Rheumatic Diseases.

[6] Allaire S, Wolfe F, Niu J, LaValley MP, Zhang B, Reisine S (2009). Current risk factors for work disability associated with rheumatoid arthritis: Recent data from a US national cohort. Arthritis and Rheumatism.

[7] Katchamart W, Narongroeknawin P, Chanapai W, Thaweeratthakul P (2019). Health-related quality of life in patients with rheumatoid arthritis. BMC Rheumatology.

[8] McBeth J, Dixon WG, Moore SM, Hellman B, James B, Kyle SD, Lunt M, Cordingley L, Yimer BB, Druce KL (2022). Sleep disturbance and quality of life in rheumatoid arthritis: Prospective mHealth study. Journal of Medical Internet Research.

[9] López-Medina C, Ramiro S, van der Heijde D, Sieper J, Dougados M, Molto A (2019). Characteristics and burden of disease in patients with radiographic and non-radiographic axial spondyloarthritis: A comparison by systematic literature review and meta-analysis. RMD Open.

[10] Rosenbaum JT, Pisenti L, Park Y, Howard RA (2019). Insight into the quality of life of patients with ankylosing spondylitis: Real-world data from a US-based life impact survey. Rheumatology and Therapy.

[11] Sieper J, Holbrook T, Black CM, Wood R, Hu X, Kachroo S (2016). Burden of illness associated with non-radiographic axial spondyloarthritis: A multiperspective European cross-sectional observational study. Clinical and Experimental Rheumatology.

[12] Gerhold K, Richter A, Schneider M, Bergerhausen HJ, Demary W, Liebhaber A, Listing J, Zink A, Strangfeld A (2015). Health-related quality of life in patients with long-standing rheumatoid arthritis in the era of biologics: Data from the German biologics register RABBIT. Rheumatology (Oxford, England).

[13] McWilliams DF, Walsh DA (2016). Factors predicting pain and early discontinuation of tumour necrosis factor-α-inhibitors in people with rheumatoid arthritis: Results from the British Society for Rheumatology Biologics Register. BMC Musculoskeletal Disorders.

[14] Minnock P, FitzGerald O, Bresnihan B (2003). Women with established rheumatoid arthritis perceive pain as the predominant impairment of health status. Rheumatology (Oxford, England).

[15] Taylor P, Manger B, Alvaro-Gracia J, Johnstone R, Gomez-Reino J, Eberhardt E, Wolfe F, Schwartzman S, Furfaro N, Kavanaugh A (2010). Patient perceptions concerning pain management in the treatment of rheumatoid arthritis. Journal of International Medical Research.

[16] Wassenberg S, Rau R, Steinfeld P, Zeidler H (2005). Very low-dose prednisolone in early rheumatoid arthritis retards radiographic progression over two years: A multicenter, double-blind, placebo-controlled trial. Arthritis and Rheumatism.

[17] Mogard E, Olofsson T, Bergman S, Bremander A, Kristensen LE, Olsen JK, Wallman JK, Lindqvist E (2021). Chronic pain and assessment of pain sensitivity in patients with axial spondyloarthritis: Results from the SPARTAKUS cohort. The Journal of Rheumatology.

[18] Zautra AJ, Parrish BP, Van Puymbroeck CM, Tennen H, Davis MC, Reich JW, Irwin M (2007). Depression history, stress, and pain in rheumatoid arthritis patients. Journal of Behavioral Medicine.

[19] Odegård S, Finset A, Mowinckel P, Kvien TK, Uhlig T (2007). Pain and psychological health status over a 10-year period in patients with recent onset rheumatoid arthritis. Annals of the Rheumatic Diseases.

[20] Günaydin R, Göksel Karatepe A, Çeşmeli N, Kaya T (2009). Fatigue in patients with ankylosing spondylitis: Relationships with disease-specific variables, depression, and sleep disturbance. Clinical Rheumatology.

[21] Deodhar A, Gensler LS, Magrey M, Walsh JA, Winseck A, Grant D, Mease PJ (2019). Assessing physical activity and sleep in axial spondyloarthritis: Measuring the gap. Rheumatology and Therapy.

[22] Latocha KM, Løppenthin KB, Østergaard M, Jennum PJ, Christensen R, Hetland M, Røgind H, Lundbak T, Midtgaard J, Esbensen BA (2020). Cognitive behavioural therapy for insomnia in patients with rheumatoid arthritis: Protocol for the randomised, single-blinded, parallel-group Sleep-RA trial. Trials.

[23] Heiberg T, Kvien TK (2002). Preferences for improved health examined in 1,024 patients with rheumatoid arthritis: Pain has highest priority. Arthritis and Rheumatism.

[24] Lee S, Oh H, Kim S, Park W, Kwon S, Lim MJ, Jung KH, Seo W (2022). Factors that influence sleep disturbance and the mediating effects of depression on sleep disturbance in patients with rheumatoid arthritis. Orthopaedic Nursing.

[25] Strand V, Singh JA (2017). Patient burden of axial spondyloarthritis. Journal of Clinical Rheumatology.

[26] Sariyildiz MA, Batmaz I, Bozkurt M, Bez Y, Cetincakmak MG, Yazmalar L, Ucar D, Celepkolu T (2014). Sleep quality in rheumatoid arthritis: Relationship between the disease severity, depression, functional status and the quality of life. Journal of Clinical Medicine Research.

[27] Boeselt T, Koczulla R, Nell C, Beutel B, Guenter K, Cassel W, Hildebrandt O, Koehler U, Kroenig J (2019). Sleep and rheumatic diseases. Best Practice & Research Clinical Rheumatology.

[28] Coskun Benliday I (2018). Sleep impairment: An obstacle to achieve optimal quality of life in rheumatoid arthritis. Rheumatology International.

[29] Editorial. (2022). A wake-up call for sleep in rheumatic diseases. The Lancet Rheumatology.

[30] Li Y, Zhang S, Zhu J, Du X, Huang F (2012). Sleep disturbances are associated with increased pain, disease activity, depression, and anxiety in ankylosing spondylitis: A case-control study. Arthritis Research & Therapy.

[31] Nicassio PM, Ormseth SR, Kay M, Custodio M, Irwin MR, Olmstead R, Weisman MH (2012). The contribution of pain and depression to self-reported sleep disturbance in patients with rheumatoid arthritis. The Journal of Pain.

[32] Finan PH, Goodin BR, Smith MT (2013). The association of sleep and pain: An update and a path forward. The Journal of Pain.

[33] Irwin MR, Olmstead R, Carrillo C, Sadeghi N, Fitzgerald JD, Ranganath VK, Nicassio PM (2012). Sleep loss exacerbates fatigue, depression, and pain in rheumatoid arthritis. Sleep.

[34] Minnock P, Veale DJ, Bresnihan B, FitzGerald O, McKee G (2015). Factors that influence fatigue status in patients with severe rheumatoid arthritis (RA) and good disease outcome following 6 months of TNF inhibitor therapy: A comparative analysis. Clinical Rheumatology.

[35] Rohleder N, Aringer M, Boentert M (2012). Role of interleukin-6 in stress, sleep, and fatigue. Annals of the New York Academy of Sciences.

[36] De Cock D, Doumen M, Vervloesem C, Van Breda A, Bertrand D, Pazmino S, Westhovens R, Verschueren P (2022). Psychological stress in rheumatoid arthritis: A systematic scoping review. Seminars in Arthritis and Rheumatism.

[37] Liu Y, Ho RC, Mak A (2012). The role of interleukin (IL)-17 in anxiety and depression of patients with rheumatoid arthritis. International Journal of Rheumatic Diseases.

[38] Mease PJ, Liu M, Rebello S, Kang H, Yi E, Park Y, Greenberg JD (2019). Comparative disease burden in patients with rheumatoid arthritis, psoriatic arthritis, or axial spondyloarthritis: Data from two Corona registries. Rheumatology and Therapy.

[39] Palinkas LA, Horwitz SM, Green CA, Wisdom JP, Duan N, Hoagwood K (2015). Purposeful sampling for qualitative data collection and analysis in mixed method implementation research. Administration and Policy in Mental Health and Mental Health Services Research.

[40] Creswell JW, Poth CN (2017). Qualitative inquiry and research design: Choosing among five approaches.

[41] Patton MQ (2014). Qualitative research &amp; evaluation methods: Integrating theory and practice.

[42] Patrick DL, Burke LB, Gwaltney CJ, Leidy NK, Martin ML, Molsen E, Ring L (2011). Content validity–establishing and reporting the evidence in newly developed patient-reported outcomes (PRO) instruments for medical product evaluation: ISPOR PRO good research practices task force report: Part 1–eliciting concepts for a new PRO instrument. Value in Health.

[43] Braun V, Clarke V (2006). Using thematic analysis in psychology. Qualitative Research in Psychology.

[44] Vaismoradi M, Turunen H, Bondas T (2013). Content analysis and thematic analysis: Implications for conducting a qualitative descriptive study. Nursing &amp; Health Sciences.

[45] Vollstedt, M., & Rezat, S. (2019). An introduction to grounded theory with a special focus on axial coding and the coding paradigm. In G. Kaiser & N. Presmeg (Eds.), *ICME-13 Monographs. Compendium for early career researchers in mathematics education* (pp. 81–100). Springer, Cham.

[46] Turner-Bowker DM, Lamoureux RE, Stokes J, Litcher-Kelly L, Galipeau N, Yaworsky A, Solomon J, Shields AL (2018). Informing a priori sample size estimation in qualitative concept elicitation interview studies for clinical outcome assessment instrument development. Value in Health.

[47] Lee YC, Lu B, Edwards RR, Wasan AD, Nassikas NJ, Clauw D, Solomon DH, Karlson E (2013). The role of sleep problems in central pain processing in rheumatoid arthritis. Arthritis and Rheumatism.

[48] Chowdhury T, Dutta J, Noel P, Islam R, Gonzalez-Peltier G, Azad S, Shankar M, Rayapureddy AK, Deb Roy P, Gousy N, Hassan KN (2022). An overview on causes of nonadherence in the treatment of rheumatoid arthritis: Its effect on mortality and ways to improve adherence. Cureus.

[49] Oliver JE, Silman AJ (2009). Why are women predisposed to autoimmune rheumatic diseases?. Arthritis Research & Therapy.

[50] Troxel WM (2022). Sleep health among adolescents and adults during the COVID-19 pandemic: Introduction to the special issue. Behavioral Sleep Medicine.

[51] Shan D, Liu C, Li S, Zheng Y (2022). Increased anxiety from fear of Omicron in China as compared to North America and Western Europe: A cross-sectional Kendall's tau-b analysis using the generalized anxiety disorder 7-item questionnaire. Frontiers in Psychiatry.

[52] Ebrahimi OV, Hoffart A, Johnson SU (2021). Physical distancing and mental health during the COVID-19 pandemic: Factors associated with psychological symptoms and adherence to pandemic mitigation strategies. Clinical Psychological Science.

[53] Mazza C, Ricci E, Biondi S, Colasanti M, Ferracuti S, Napoli C, Roma P (2020). A nationwide survey of psychological distress among Italian people during the COVID-19 pandemic: Immediate psychological responses and associated factors. International Journal of Environmental Research and Public Health.

[54] Wang C, Pan R, Wan X, Tan Y, Xu L, McIntyre RS, Choo FN, Tran B, Ho R, Sharma VK, Ho C (2020). A longitudinal study on the mental health of general population during the COVID-19 epidemic in China. Brain, Behavior, and Immunity.

