# Supplementary material for: A concept elicitation study to understand the relationship between sleep and pain in rheumatoid arthritis and axial spondyloarthritis
Source: Qual Life Res. 2023 Oct 27;33(2):373–85. doi: 10.1007/s11136-023-03524-9 (PMC10850285; doi:10.1007/s11136-023-03524-9)
Supplement: Supplementary file 1 — Supplementary file1 (DOCX 35 KB) [file 11136_2023_3524_MOESM1_ESM.docx]

**Supplementary information**

**A concept elicitation study to understand the relationship between sleep and pain in rheumatoid arthritis and axial spondyloarthritis**

Kimberly Raymond^1^, Wen-Hung Chen^2^, Marguerite Bracher^3^, April Foster^1^, Andrew Lovley^1^, Cory Saucier^1^, Kristi Jackson^1^, Eleanor J. McDermott^4^

*^1^QualityMetric Incorporated, Johnston, RI, USA*

*^2^GSK, Global Value Evidence & Outcomes, Collegeville, PA, USA*

*^3^GSK, Global Value Evidence & Outcomes, Stevenage, Hertfordshire, UK*

*^4^GSK, Real-World Study Delivery, Stevenage, Hertfordshire, UK*

**Corresponding author:**

Wen-Hung Chen

Full address: 1250 South Collegeville Road, Building 4, 4^th^ floor, Collegeville, PA 19426, USA

Tel: +1 610-917-2833

Email: [wen-hung.x.chen@gsk.com](mailto:wen-hung.x.chen@gsk.com)

**Supplementary Table 1** Quotes provided by participants in describing their experience of sleep and sleep quality, pain, fatigue, and emotional health

| **Sleep** | |
| --- | --- |
| *RA* | |
| “I’m in the bed for at least eight or nine hours, but I probably only sleep five or six hours, because the rest of the time I’m turning – I’m uh, twisting and turning, and something feels uncomfortable, or I don’t know. I just – I don’t sleep very well.” (Pt 04, RA) | “Okay, total hours, I would say – um, well, it’s- a - attempt to go to sleep. I guess, on average, I try about seven hours. But what you - you look at my - my, uh, Apple watch and I don’t get that. I usually get on average maybe about four and a half to five.” (Pt 02, RA) |
| “Typically, when I’m ready for bed, I’m washed up, I brush my teeth, I lay down and um, I’m asleep before I hit the pillow. Really pretty exhausted, so it’s not like uh … I might lose a little sleep. I might discuss with my wife some family business or something before I sleep, but I don’t, I don’t get stressed out. I’m like, asleep before she is. So typically, yeah, I’m laying down and falling asleep. My head hits the pillow, I’m done.” (Pt 09, RA) | “In the mornings, I’m really tired…Sometimes, I feel like, no, I don’t get enough sleep. I should be sleeping more, or better, or something… I probably need to sleep a little more, I’m sure.” (Pt 04, RA) |
| “Um, poor [quality]. I’m just not comfortable, and –and if I lay on one side then that side will start hurting and then I have to lay over or, you know, it – it – you – I don’t know if my body crunches up or what it is, and then I need to kind of stretch it out a little bit to try to get it so that it – it doesn’t feel so cramped or crumpled up, um, yeah. It’s just hard – it’s not a good thing at all for me. At all.” (Pt 08, RA) | “Last night, the same as any other night. I probably – well, I know I was in bed before 9:00. So, I didn’t, like, turn over to go to sleep at 9:00. But I was in bed by 9:00. By 10:00, I’m sure I was asleep. And again, I’m sure I slept until about 12:00 – about midnight or so, and then I started tossing and turning. Because, oh, my shoulder feels uncomfortable, my elbow hurts  – something, you know, wakes you up, and you’re just – you try to get in a comfortable  position, and then maybe you're awake. I don’t know how long I’m awake – maybe, I don’t know, 15, 20, 30 minutes. You fall back asleep, you sleep a little bit for maybe another hour, and then you wake up again and it’s the same thing over, until you actually get up in the morning.” (Pt 04, RA) |
| *axSpA* | |
| “So, um, I am, I’m in the bed for eight hours. Um, but as far as like sleep where I, I know that I’m asleep, it’s maybe five of those, I would guess, um, because I wake often, um, yeah. I mean, where I’m aware of it, and then I fall back asleep.” (Pt 08, axSpA) | “Interrupted sleep is certainly not as good, you know. Sometimes I don’t get that much  sleep, but if it’s interrupted by pain and discomfort, then you have the problem of getting back into a good sleep again. So, a lot of times I will wake up and feel like I’m not fully rested because the different stages of sleep have been interrupted and I have to start from scratch.” (Pt 03, axSpA) |
| **Pain** | |
| *RA* | |
| “It’s in different locations. You can feel it over your entire body, but usually it’s symmetrical. So, whatever side – if your elbow is hurting on the right side, then your knee is going to be hurting – you know, it’s like that. It doesn’t – it’s not like one thing on this side and one thing on that side. It’s – it’s all the way down.” (Pt 04, RA) | “You know, maybe my mobility has something to do with it and, uh, that keeps me, I don’t know, in more pain or just, you know, just being not able to move around as much as I had been.” (Pt 03, RA) |
| “The mornings when I try to walk daily and the more I do – the more physical activities I do the worse I feel – w-which stinks. [Laughter] ‘cause they say with rheumatoid arthritis you should try to still be active or whatever, but then I don’t feel as good; and like even the bottom of my feet – if I walk too long or on my feet too long, the bottom, um, starts to burn. The pain is like a burning sensation.” (Pt 05, RA) | “It’s definitely different. Because I’m having a raspy voice. I have dry mouth. My eyes are watering. My ears feel like they’re like trying to fall off… When I say fall off, it’s getting very scratch in the drum, and I know that I’m not sick or you know, that-that’s a flare-up… When I’m getting a lot of knee pain, both knees are hurting… My toes are im-in-in-a, you know, inflamed… Or pain around um, the front of my feet or the bottom of my feet and-and walking… Normal pain like with every other day, you’re just going to get a little bit of p-pain  around joints and the inflamed area. And not having um, ex-excessive amount of pain for several days.” (Pt 12, RA) |
| *axSpA* | |
| “[The pain] varies… Sometimes, you know, I—I feel fine, there’s no symptoms. Uh, but  there’s other times where it’s just, uh, you know, uh, very debilitating, where I can’t even move… if I take, like, some—like an Aleve or an Advil, it’s, you know, going to—gets rid of the painful feeling. So, sometimes I’ll do that. Um, that can kind of, you know, make me feel better, at least temporarily. But yes, it generally happens—generally happens at least a couple times a week.” (Pt 06, axSpA) | “In the back, it feels like an ache as well. It's not – I never experienced super sharp pain  unless it's like going – it's starting to travel to other areas. I can like vividly feel it. Uhm, it feels dull in my back as well. A dull ache, but, uhm, more like I have to like crack my back or like push in certain spots.” (Pt 07, axSpA) |
| “For me, it’s really I just got the tightness in my hips. It hurts to walk. Uh, the pain that  radiates basically from the middle, the mid – my midback, all the way down my legs.” (Pt 1, axSpA) | “And I couldn't get out of bed, uhm, it will – I don't usually – usually my pain just affects me falling asleep, but it woke me out of my sleep. And I, uhm, I couldn't get out of bed. I couldn't walk to the bathroom. I called my mom – To come help me. And then, I had to spend all day in bed. Because it just was so unbearable to even like – it hurt to be in bed. But it was the only place that I wasn't like pushing it.” (Pt 07, axSpA) |
| **Fatigue** | |
| *RA* | |
| “I experience it [fatigue] every single day, but some days it might be like, down on a level three; other days it could be up to eight or nine.” (Pt 14, RA) | “I feel like my RA manifests in the afternoon where I’ve run out of steam, and then I just need to relax. About 4:00. Sometimes a ittle earlier. Sometimes like 2:00-2:30, but mostly that 4:00 mark is like my body sends me a message.” (Pt 11, RA) |
| “God, it so varies, it’s so hard for me to, uh, give you a – a set – I mean, it could be a couple times a week to, like, five times a week. You know what I mean? It just depends on – it depends on what I’ve done that week.” (Pt 02, RA) | “It gets worse if I’m more active. Um you know, like I said, I have to, I have to like, you know, take the garbage out. I have to, you know, keep up with the house. I have to go up and down the stairs. And by the end of the day, I just sit on the couch because it hurts. You know, my joints will hurt, and um I’m also fatigued.” (Pt 10, RA) |
| “Otherwise, it’s 120 degrees, so regardless of if I’m in my car and I’ve got the air  conditioning on, once I step outside that car, the heat drains me.” (Pt 03, RA) | “I notice there are times where I’ll have a bout of anywhere from two wee – two days to a week or whatever where I’m feeling extremely fatigued and tired and have tracked that the arthritis is more bothersome during that. Like, like I guess they used to call that – I don’t know if they still do, and I – I - that I’m having a flare up.” (Pt 01, RA) |
| *asSpA* | |
| “I’ve known tiredness, it seems like tiredness is not the same as fatigue. Fatigue is more when your whole body is, is kind of shutting down. And tiredness is when you need rest and sleep, and it’s a different thing than fatigue” (Pt 03, axSpA). | “The fatigue is there like it's kind of maybe like three or four days a week. Only when – Yeah like, about three times – three or four times a week.” (Pt 10, axSpA) |
| “Yes, I’d say daily. It is always there with me. Some days it’s worse than others. And it seems like if I try to do anything that is strenuous, I don’t last as long.” (Pt 03, axSpA) | “I feel really fatigued – I don't know, it don't hit me as the day is going it's like I'm in  overdrive like I'm constantly moving or whatever but when I stop, I can really – it really hits me.” (Pt 10, axSpA) |
| “Yesterday, the weather came in and, uhm, I, I made it through the morning. And then come, I want to say noon or one o’clock or something like that, I had to go and lay down. I didn’t have a choice, and I, I slept for maybe 30 minutes, but I laid down for about two hours. Uhm, yeah, I just couldn’t move or function or think.” (Pt 11, axSpA) |  |
| **Emotional health** | |
| *RA* | |
| “Like the anxiety part of it, it’s a physiological thing that I learned about my body that when I’m experiencing like, it’s not like I’m anxious, like oh my god, I’m going to die. It literally is a physiological reaction to my body being like either severe pain or sick. It just happens.” (Pt 01, RA) | “No, I-I try, you know, I uh, emotionally, I think that I’m in a good place right now. I uh, you know, you know, I don’t have too many things that upset me or anything. I just um, I have, you know, I have accepted that I have RA. I accepted that a long time ago and I accepted the treatment that, um, they prescribed for me.” (Pt 06, RA) |
| “I think – they told me early on that stress is a major factor, um, for autoimmune diseases period. Um, that’s even possibly what brings them on – just they don’t really know. Um, but I do think in stressful situations – and, especially with everything we’ve had going on this year, um, with COVID and everything, because this year has probably been worse for my RA  than I could say last year was.” (Pt 04, RA) |  |
| *axSpA* | |
| “Yeah, because it’s a stress. I’m already stressed. Stress probably causes inflammation, I don't know but yeah and none – none of this crap was really going on when it all first began, at least – at least not to this level, let’s put it that way but it is now.” (Pt 05, axSpA) | “Yes, I think, I think that, I-I think our bodies react to pain by tensing up and I think that we also react to emotional upsets by having pain. I don’t know if you’ve ever noticed it, but if I get upset about something, I can notice that my neck and shoulders tighten up. So, I think it’s, I think yeah, relaxation has a lot to do with it.” (Pt 03, axSpA) |

*axSpA* axial spondyloarthritis, *Pt* patient, *RA* rheumatoid arthritis

**Supplementary Table 2** Quotes provided by participants with RA in relation to identified themes

| **Theme** | **Example quotes** |
| --- | --- |
| Theme 1: Pain as a primary driver of sleep disturbance, affecting both falling asleep and night-time waking | “If I hurt I – I – I have a hard time going to sleep because of the ache and the hurt and it – I just can’t go to sleep” (Pt 08, RA) |
| Falling asleep | “I guess it would be the severity of the pain, like if it’s a bad pain night, then yeah, then it – it’s – it’s – it will be longer to fall asleep. And I’ll toss and turn and like I said try – I can’t find a spot for myself” (Pt 01, RA) |
| Staying asleep | “It was like a stabbing pain, you know. It was like, like something would just jab at you and just, and – and – and when I would go to sleep, I might sleep about an hour and a half and it would just jab at me. Ans wake me up” (Pt 06 RA) |
| Sleep quality | “Most times it’s just the pain that doesn’t let me sleep well” (Pt 05 RA) |
| Sleep quantity | “I never seem to sleep longer um than three hours. And then I’ll wake up for a while, and then I might go back to sleep for another two. I would say I never have uninterrupted sleep and um probably the longest I sleep in a, you know, as far as consecutively, it would probably be three hours. Total amount of sleep would probably be six to seven hours. But it’s never consecutive. You know, it’s always broken up” (Pt 10 RA) |
| Theme 2: A bi-directional relationship whereby pain worsens sleep disturbance, and sleep disturbance further aggravates pain | “I think they’re all related. I think that each one of them plays a role and if for one, I’m not sleeping well then I’d say it’s going to impact my emotions and probably my pain for the day the next day or even if the pain is impacting my life it’s going to affect my emotions and my sleep because I’m not going to be able to sleep and then all I’m trying to do is not hurt, so that’s going to affect my emotions, too. But I think they all intermix” (Pt 08, RA) |
| Theme 3: Other factors influencing sleep disturbance |  |
| 3a: Sleep disturbances due to causes other than pain (e.g., anxiety, fatigue) | “Then, the other, is just kind of the mental drag of having RA has on you too, Um, I think it’s those things that probably impact my sleep, um, when it’s not pain more than anything” (Pt 04 RA) |
|  | “You know, like I said, just the panic anxiety and getting old and having to get up and pee” (Pt 04 RA) |
|  | “Sometimes I can just be overly tired, and it makes it hard to go to sleep because you just get overly tired. You just get so exhausted that you think, oh this is awesome – I’m just so wiped out I’m just going to go home and I’m going to crash. And you get your body – you just get yourself to the point where you are just overly tired you can’t sleep” (Pt 02 RA) |
| 3b: Sleep disturbances due to causes likely related to disease activity (e.g., stiff, locking joints) or other known aspects related to disease management (e.g., medication) | “Well, I guess I Could say like sometimes, like the finger, like I think, you know like my, the – the, how all of a sudden, they’ll like, lock up? It’s not painful, I get that in the right ankle sometimes where like, it’s not pain, but if I have it in the wrong position, all of a sudden, it’ll like, I can’t move my ankle. So that’ll impact it, and then I’ll kind of have to reposition, and then it’s like, it – it all kind of comes back to that can’t find a comfortable spot, I can’t find yeah. And so, it’s not like pain, but it definitely is, you know, related to the RA” (Pt01 RA) |
|  | “I noticed like, my legs were really stiff and they were swelling and I just felt so…I was tired, but I couldn’t sleep” (Pt 12 RA) |
| 3c: Sleep disturbances due to causes unrelated to disease (e.g., needing to use the restroom) or to unknown causes | “And now not always 100% it is the discomfort waking me. It’s like, you know there are times where I have to go to the bathroom, too. So, it’s not always 100% that it’s – it’s that. You know what I mean? There’s – you know, I’m 60 years old, so there are times that I have to go to the bathroom, too” (Pt 02 RA) |
|  | “Well, sometimes I have to go to the bathroom. [Laughs] You know, but, or I’ll hear a loud noise. You know, something like that” (Pt 01, RA) |
|  | “It just seems so random, like why did I sleep so well three nights ago? I wouldn’t know what I did different versus the next night, why didn’t I sleep? Or why did I, you know?” (Pt 01 RA) |
|  | “I actually think this has already changed, like, my sleeping pattern and doesn’t allow a person to sleep as they should; so, like, even if I’m not in that much pain it has altered my sleeping pattern” (Pt 05, RA) |
|  | “There are some nights that I just can’t sleep, uh, for no apparent reason, you know, I haven’t had a nap in the day, I’ve had a great day, I haven’t had, you know, over-exhausted myself from being outside, you know, so for whatever reason, whether if it’s just if it be – I can’t pinpoint it, whether it's been something heavy on my mind or I’ll be stressed about something or I’m thinking, overthinking something, for whatever reason, uh, yeah, it just, it just happens that I can’t sleep” (Pt 03 RA) |

*axSpA* axial spondyloarthritis, *Pt* patient, *RA* rheumatoid arthritis

**Supplementary Table 3** Quotes provided by participants with axSpA in relation to identified themes

| **Theme** | **Example quotes** |
| --- | --- |
| Theme 1: Pain as a primary driver of sleep disturbance, affecting both falling asleep and night-time waking | “I mean, if the pain is worse, I’ll sleep less, and my fatigue is worse the next day. It’s as simple as that, for me” (Pt 01, axSpA) |
|  | “I don’t really notice anything other than I think that, you know, the pain is what causes me to not be able to sleep. That’s the only pattern that I see which then the no sleep leads to the fatigue” (Pt 10, axSpA) |
| Falling asleep | “Well, when I, when I first go to bed – and lie down, uhm, typically, I have a hard time – it takes me a long time to fall asleep. Because lying down causes my pain level to go up. So, uhm, you know, I – it’s hard getting comfortable” (Pt 12, axSpA) |
| Staying asleep | “When I can’t seem to get comfortable, the times that I do get comfortable, I wake up during the night and I’m hurting, so it’s like, I cannot get the uninterrupted sleep that I really need because of the pain” (Pt 03, axSpA) |
| Sleep quality | “What I’ll say is usually the nights that I sleep that well, I might have one or two interruptions where I wake up to go to the bathroom or wake up because I’ve been sleeping hard in a solid position and the pain would start. Switch positions fall back to sleep and still end up being able to sleep and have kind of a normal waking time. You know, 8.00. Uhm, so, that, to me is a good restful night. And even if I’m woken up, if I can go back to sleep, you know, the key is just getting the rest for me” (Pt 14, axSpA) |
| Sleep quantity | “it changes because there are times when you know, I – I don’t have this much pain. So, like on a good day I might get like seven and a half hours of sleep. But I’d say over the course of a year, at least – at least I’d say 30 – I’d say 40% of it is – is sleep deprivation from the pain” (Pt 02, axSpA) |
| Theme 2: A bi-directional relationship whereby pain worsens sleep disturbance, and sleep disturbance further aggravates pain | “Well, I think with that one is my sleep is worse when my pain is worse, and if my pain is bad, it can trigger a depressive, an episode of depression. Um, so the next day is just going to be more of the same, more pain, more fatigue” (Pt 08, axSpA) |
| Theme 3: Other factors influencing sleep disturbance |  |
| 3a: Sleep disturbances due to causes other than pain (e.g., anxiety, fatigue) | “If I’m, you know, dealing with depression or something like that, then definitely it’s hard to go to sleep because, you know, my mind is racing, I’m having a lot of thoughts. Un, and then when I wake up, instead of, uh, falling right back asleep when I wake up immediately, you know, my mind starts going. And it could be, um, frustration that kicks in, and then, you know, my mind goes to this different place, um, about, you know, either the pain or whatever it is, um, which makes it a little bit harder to fall back asleep2 (Pt 08, axSpA) |
|  | “On the days where I’m extra fatigued during the day, I can actually see that my energy level goes up at night so for some reason, my body’s so weird like that. Like, um, it will go up and night and then I’ll think – I’ll keep thinking like oh, well I was tired during the day, why am I not tired now? And then my brain will get in the way of me going to bed because like I’m confused by my body’s reaction” (Pt 04, axSpA) |
| 3b: Sleep disturbances due to causes likely related to disease activity (e.g., stiff, locking joints) or other known aspects related to disease management (e.g., medication) | “It takes a while for the muscles in my back and everything to kind of relax and get to a place that I can get comfortable. Uhm, so it just takes time, I guess, for my body to get into a position that I don’t feel like, tight and uncomfortable as I do normally” (Pt 13, axSpA) |
|  | “The pain has woken me up. Or it’s stiffness and I’ve gotten kind of crunchy in this position2 (Pt 14, axSpA) |
| 3c: Sleep disturbances due to causes unrelated to disease (e.g., needing to use the restroom) or to unknown causes | “I might wake up like once to go to the bathroom during the night, that’s about it” (Pt 02, axSpA) |
|  | “There are other factors that wake me up. Uhm, my daughter is almost two years old, and she’s just gotten to the point where she can get out of bed herself, so she’ll wake up in the middle of the night and, and come and wake one of us up, me, me or my wife. Uhm, so that’s, that’s been happening recently. Uhm, sometimes, I live in a city, so sometimes there will be a noise, or, uhm, my wife snoring, or, you know, something that will wake me up” (Pt 11, axSpA) |
|  | “I wake up sometimes in the middle of the night. Uhm, I don’t know why I wake up” (Pt 07, axSpA) |
|  | “I do have those nights. That’s, you know, you can’t really say it’s because of the pain, but it is, I think it’s related to this condition that you have sleepless nights even without having a lot of discomfort” (Pt 03, axSpA) |

*axSpA* axial spondyloarthritis, *Pt* patient, *RA* rheumatoid arthritis
